# Study on the adsorption of nitrogen and phosphorus from biogas slurry by NaCl-modified zeolite

**DOI:** 10.1371/journal.pone.0176109

**Published:** 2017-05-19

**Authors:** Qunpeng Cheng, Hongxia Li, Yilu Xu, Song Chen, Yuhua Liao, Fang Deng, Jianfen Li

**Affiliations:** 1 School of Chemical and Environmental Engineering, Wuhan Polytechnic University, Wuhan, PR China; 2 Global Centre for Environmental Remediation, Advanced Technology Centre, Faculty of Science and Information Technology, The University of Newcastle, Callaghan, New South Wales, Australia; 3 School of Environmental Science and Engineering, HuaZhong University of Science and Technology, Wuhan, PR China; Southwest University, CHINA

## Abstract

A NaCl-modified zeolite was used to simultaneously remove nitrogen and phosphate from biogas slurry. The effect of pH, contact time and dosage of absorbants on the removal efficiency of nitrogen and phosphate were studied. The results showed that the highest removal efficiency of NH_4_^+^-N (92.13%) and PO_4_^3−^-P (90.3%) were achieved at pH 8. While the zeolite doses ranged from 0.5 to 5 g/100 ml, NH_4_^+^-N and PO_4_^3−^-P removal efficiencies ranged from 5.19% to 94.94% and 72.16% to 91.63% respectively. The adsorption isotherms of N and P removal with NaCl-modified zeolite were well described by Langmuir models, suggesting the homogeneous sorption mechanisms. While through intra-particle diffusion model to analyze the influence of contact time, it showed that the adsorption process of NH_4_^+^-N and PO_4_^3−^-P followed the second step of intra-particle diffusion model. The surface diffusion adsorption step was very fast which was finished in a short time.

## Introduction

Piggery wastewater is well known for its high concentration of organic matters(OM), nitrogen (N) and phosphorus (P)[1,2]. Because of low biomass production and low treatment cost, Anaerobic digestion had been widely used as an efficiency treatment. While biogas slurry are still rich in nitrogen (NH_4_^+^-N > 400 mg/L) and phosphorus (PO_4_^3−^-P >100 mg/L)[3,4]. Usually biogas slurry are directly used to irrigate the land filed directly to improve soil fertility and consequently be bebeficial to crop growth [5]. Meanwhile, excessive land application will lead nutrient loss from soil to water through runoff and leaching which could cause adverse consequences to groundwater[6,7]. Therefore, an effective and economical method to removal of N and P from biogas slurry is necessary.

Instead land application, adsorption is considered to be a simple and effective technique for the removal of nutrients from the wastewater. To remove organic contaminations from wastewater, some commonly used adsorbents are activated carbon[8,9], clay minerals [10,11], chemical amendment[12,13], biochar[14,15], and zeolites[16–18]. Among those adsorbents, zeolites are widely applied for the purpose of reusing the effluent water, reducing pollution of water resources, reducing gas emissions through modifying the physiochemical properties of manure and decreasing water consumption[19–21]. An Australian zeolite with iron-coating and without iron-coating were used to remove Pb, Cu, Cd, Cr and Zn from aqueous solutions in batch and column experiments. Results showed that with pH 6.5, the Langmuir adsorption capacities of those five heavy metals ranged from 5.0–11.2 mg/g for single metal, while 3.7–7.6 mg/g for mixed metals solution [22]. Similar results were obseved by Egashira et al. They used Mongolian natural zeolites to adsorb Cu, Zn and Mn from model aqueous wastewater (pH 3–5), and found out that the adsorption capacities of 8.32–10.24 mg/g for Cu, 9.1–54.6 mg/g for Zn, 6.05–11.05 mg/g respectively for Mn [23]. Lin et al studied the effect of natural zeolite on the removal of P and NH_4_^+^-N from orthophosphate and ammonium-nitrogen laden wastewaters at pH 3–11 in batch and continuous tests. The results showed that the highest removal rate (98.9% for P and 68.9% for NH_4_^+^-N) was reached at pH 9.3[24]. Chen et al.used nano-zeolites synthesized from fly ash (ZFA) was used to simultaneously remove ammonium (N) and phosphate(P) in anaerobically digested swine wastewater [25]. N and P removal efficiencies ranged from 41% to 95% and 75% to 98%, respectively with a range of ZFA doses from 0.25 to 8 g/100 mL. The adsorption capacity is related to the wastewater properties as well as the colloidal properties and negatively-charged layers of zeoloties.

By evaluation parameters such as pH, adsorbent dosage and initial concentration of zeolite,the aim of this work was to investigate the impact of NaCl-modified zeolite on the removal of nitrogen and phosphorus from biogas slurry. The adsorption isotherms were adjusted to the models of Langmuir and Freundlich. Kinetic models of adsorption were used to analyze the kinetics and the zeolite adsorption mechanisms on the adsorbents.

## Materials and method

My study did not involve human participants, specimens or tissue samples, or vertebrate animals, embryos or tissues:

1. We state clearly that no specific permissions were required for these locations/activities, and provide details on why this is the case;
2. We confirm that the field studies did not involve endangered or protected species.
3. We confirm that the authors had received approval from the COFCO Corporation to collect samples from the pig treatment plant.

About the name and product number of the zeolite acquired from the Wuhan rhyme siphon water treatment material limited company was the natural zeolite where there were no specific product number. They sell the zeolite in bulk and we just obtained some from them to do the research.

The natural zeolite used in this research was obtained from Wuhan rhyme siphon water treatment material limited company in Wuhan, China. The chemical composition of the natural zeolite is given in Table 1. The particle size of the used zeolite was < 0.15 mm. The zeolite was fully washed several times with deionized water and dried for 24 h at 100°C. Natural zeolite was mixed with the NaCl solution in the ratio of 1:50 (g zeolite:mL NaCl). A magnetic stirrer was used to mix the sample mixture solution for 24 h at 150 rpm using a horizontal shaker at 30°C. Biogas slurry was collected from a pig treatment plant built by COFCO Corporation, located in Xinzhou, Wuhan, China (114°77´44.41"E, 30°54´12.84"N). Table 2 presents the chemical composition of the biogas slurry used in the experiment. The samples of biogas slurry were stored at 4°C until utilized.

**Table 1:** The chemical composition of zeolite (n = 3, SD).

Components	SiO_2_	Al_2_O_3_	Fe_2_O_3_	K_2_O	CaO	MgO	Na_2_O	other
wt%	69.58±1.30	12.20±0.50	0.87±0.08	1.13±0.05	2.59±0.20	0.13±0.02	2.59±0.10	10.91±0.13

**Table 2:** The charateristic of biogas slurry (n = 3, SD).

Item	pH	COD_Cr_ (mg/L)	NH_3_-N (mg/L)	PO_4_^3−^-P (mg/L)	VFA (mg/L)	EC (μs/cm)
Mean-value	8.09±0.30	2113.33±12.25	708.43±8.35	21.62±1.50	218.95±5.21	8607±15.31

The chemical composition of the zeolites was determined by energy dispersive X-ray spectros-copy (EDS) (SEA1000A, Japan). The mineralogy of the zeolite was determined using a XRD Shimadzu S6000 (Japan) diffractometer on powder samples of the zeolite. The X-ray diffractometer was equipped with a Cu target operated at 40 kV and 30 mA with a setting of 0–80° (40 min), step time 2°/min. Scanning electron microscopy (SEM), Fourier transform infrared (FTIR) spectroscopy were also inspect the structure of zeolite. TS (total solid), NH_4_^+^-N, PO_4_^3−^-P and total alkalinity (titrated to pH 4.3) were tested according the Standard Methods[26]. Batch experiments were carried out to obtain the adsorption data relative to contact time, sorptive concentration, pH, and dose of zeolite. pH was adjusted by the addition drops of strong HNO_3_/NaOH solution. Then kept constant during the whole adsorption experiments. Experiments used to determine the equilibrium time were performed with the contact time between adsorbent and adsorbate in the range from 10 min to 24 h, at pH 8.1, with an initial concentration of NH_4_^+^-N (708.4mg/L) and PO_4_^3−^-P(21.62mg/L). The influences of adsorbent dosage (0.5, 1.0, 1.5, 2.0, 3.0, 4.0 and 5.0 g of adsorbent) and of pH (6.0, 7.0, 8.0,9.0 and 10.0) on the removal of nutrients by NaCl-zeolite were studied. All adsorption experiments were performed in triplicate. The removal rate of nitrogen and phosphorus was calculated as Eq (1):

$$\eta =\frac{C_{0}-C_{e}}{C_{0}}\times 100\%$$ (1)

Where η is the removal rate (%),C_0_ is the initial concentration of nitrogen and phosphorus (mg/L), C_e_ is the concentration of nitrogen and phosphorus at equilibrium (mg/L).

### Adsorption isotherms model

Adsorption isotherms is neccesary to describe the equilibrium relationships between the amounts of ion exchanged by zeolite and its equilibrium concentration in the solution which could be helpful for the analysis and the design of the sorption systems. In this study, two adsorption isotherms were developed by Langmuir model (Eq (2)) and Freundlich model (Eq (3)).

$$\frac{C_{e}}{q_{e}}=\frac{1}{q_{\text{max}}k_{L}}+\frac{C_{e}}{q_{\text{max}}}$$ (2)

$$\text{log}q_{e}=\text{log}k_{F}+\frac{1}{n}\text{log}C_{e}$$ (3)

Where k_L_ is the Langmuir isotherm constant (L/mg), **q**_**max**_ represent the maximum ammonium ion-exchange capacity of zeolite (mg/g). k_F_ is the Freundlich isotherm constant that indicates the maximum adsorption capacity(mg/g), 1/n is the Freundlich isotherm constant which is dimensionless. The n value indicates the degree of nonlinearity between solution concentration and adsorption; when n = 1, n < 1 and n > 1 is adsorption linear, chemical and physical process, respectively. The n values of within the range of 1–10 represent good adsorption. C_e_ is the equilibrium concentration of the adsorbed substance in the liquid phase (mg/L) and q_e_ is the constant that indicates the maximum adsorbate quantity of the adsorbent (mg/g).

### Statistical analysis

The data was analysed by SPSS 15.0 using regression analysis. The goodness of correlation was evaluated with the correlation coefficient R^2^.

## Results and discussion

### Zeolite characterization

**Fig 1:**
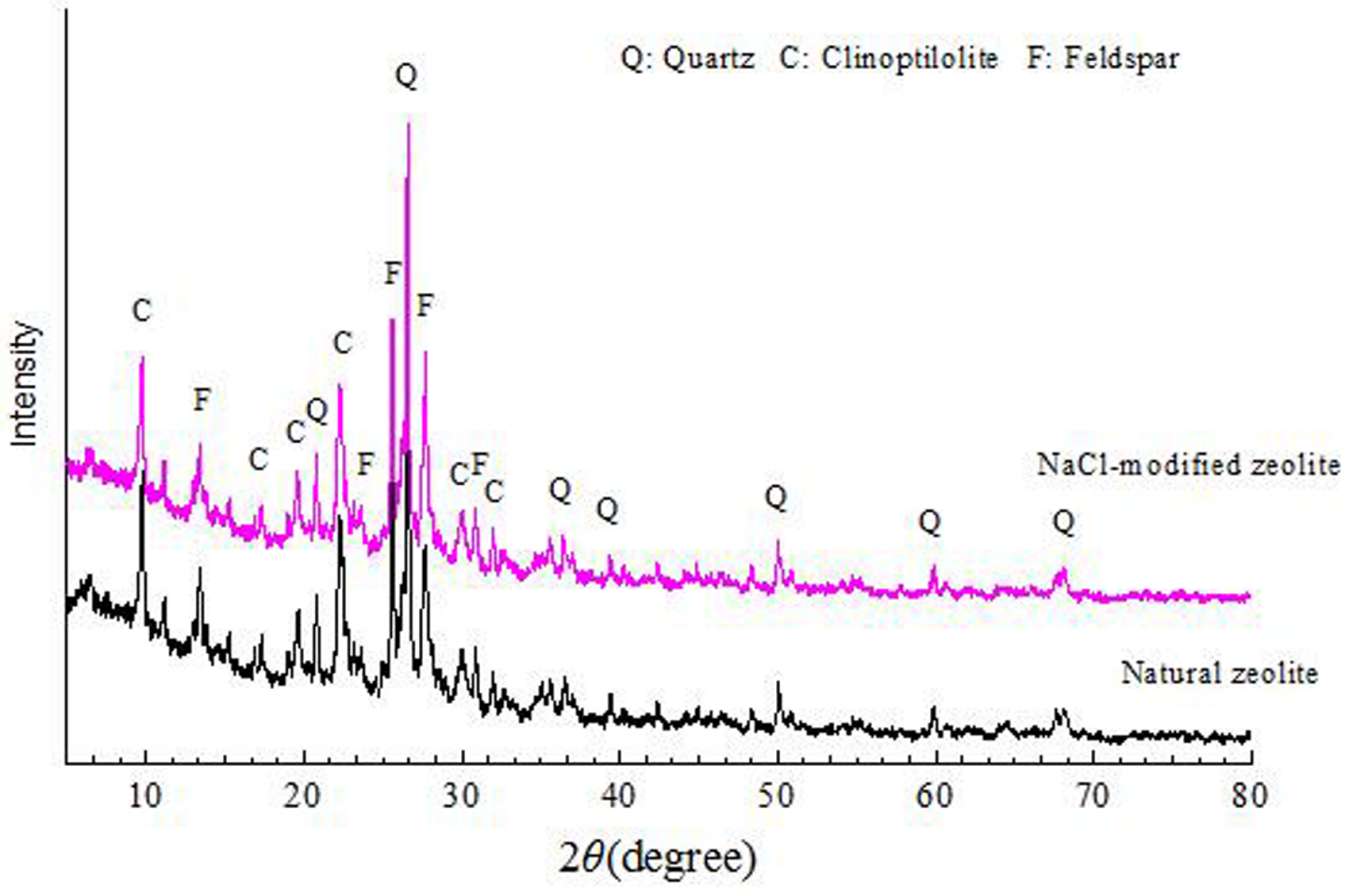
XRD patterns of the natural and NaCl-modified zeolites.

**Fig 2:**
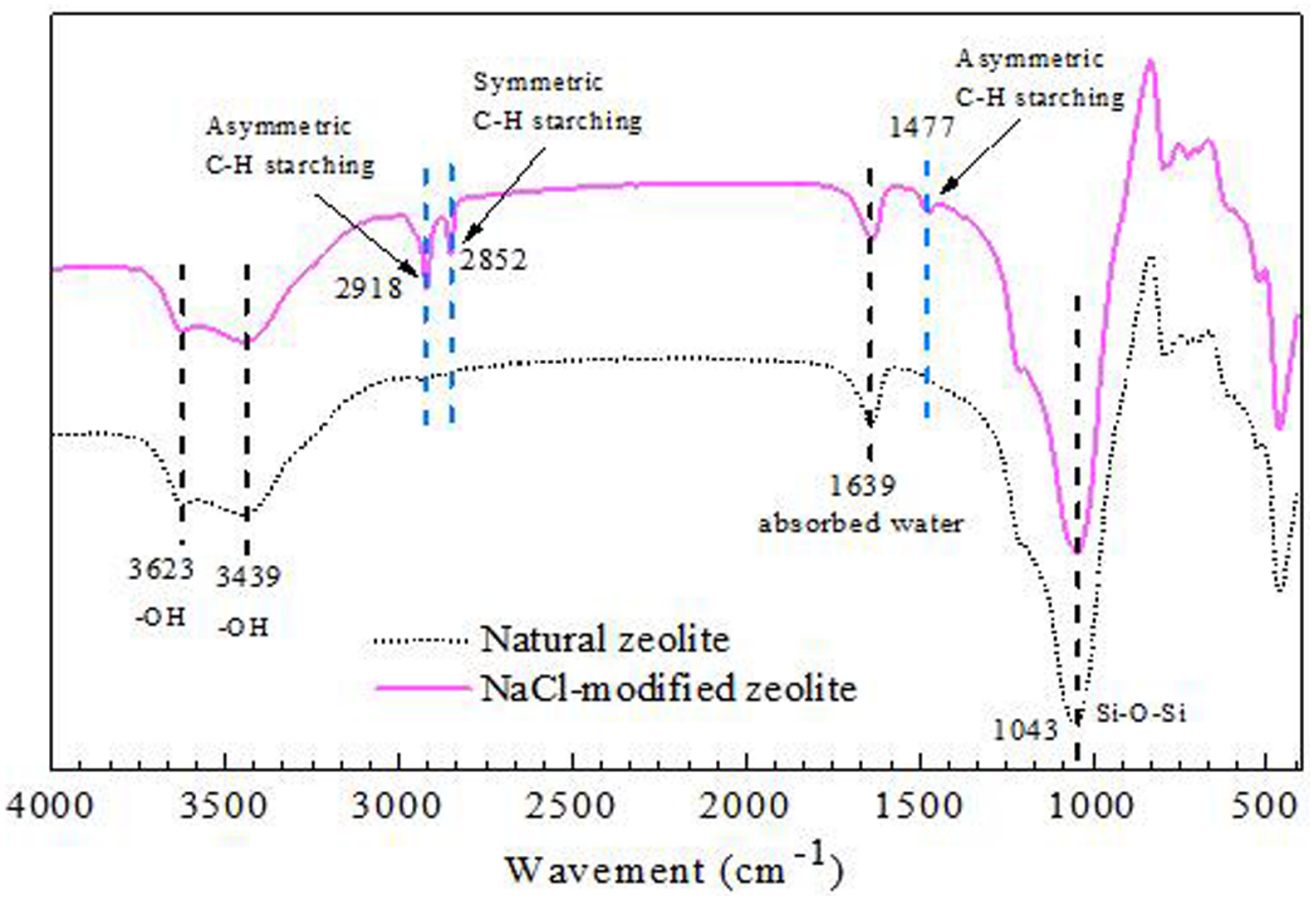
FI-IR spectra obtained for natural zeolite and NaCl-modified zeolite.

The characteristics of the nature zeolite (NZ) and modified zeolite (MZ) were displayed based on the results of XRD and XRF analyses. The XRD patterns and the elemental analysis of the NZ and MZ were shown in Fig 1 and Table 3, respectively. The XRD spectra indicated that the main mineral species of the zeolite before and after modification remained unchanged. The main composition of NZ and MZ were clinoptilolite, quartz and ferrosilite, illustrating the absence of structural degradation during modification. The content of cations like Na^+^, K^+^, and Ca^2+^ in zeolites determines the exchange ability and adsorption. The more cations Na^+^, K^+^, and Ca^2+^, the stronger the ion-exchange ability. While Si/Al ratio influences the thermal and physical stability. Zeolites with higher Si/Al exhibits a very high physico-chemical durability. The improvements of chemical compositions through modification of zeolites such as substitution of some (Si) and aluminium (Al) (or other metals) with cations Na^+^, K^+^, and Ca^2+^ lead to a negative charge on the framework which will increase the ion exchange ability of zeolites, especially for Na^+^ to the ion-exchange with NH_4_^+^. The XRF results showed that after NaCl modification, the contents of the exchangeable cations such as Al^3+^, Ca^2+^, and K^+^ decreased, while the Si^4+^, Na^+^ amount increased significantly. It demonstrated that Al^3+^, K^+^, and Ca^2+^ were replaced by Na^+^ which was benefit for the removal of the other cations especially NH_4_^+^ [27]. Fig 2 showed the FTIR spectra of NZ and MZ in the range of 500–4000 cm^-1^. The typical FTIR spectra for zeolite bands was observed at the region below 1700 cm^−1^. In this region, the bands from the zeolites backbones (Si–O–Si and Si–O–Al) which was composed of bending and stretching of Si–O–Si, Si–O and Al could be observed. Adsorption bands occurred at 3500 cm^−1^ and 1639 cm^−1^ were associated with–OH stretching and bending frequencies, respectively. A peak obtained from 2852 and 2918 cm^−1^ was significantly increased after modification with NaCl. These results appeared that the successful grafting of Na^+^ onto the surface hydroxyl groups of zeolite which was verfied by the results of XRD.

**Table 3:** Chemical composition of NZ and MZ byXRF (wt%).

Oxide	NZ	MZ
Al_2_O_3_	15.14	12.75
SiO_2_	72.85	77.58
K_2_O	1.71	1.09
CaO	2.55	0.88
TiO_2_	0.13	0.09
Na_2_O	3.53	6.04
MnO	0.05	0.04
Fe_2_O_3_	0.56	0.58
others	3.48	1.13

**Fig 3:**
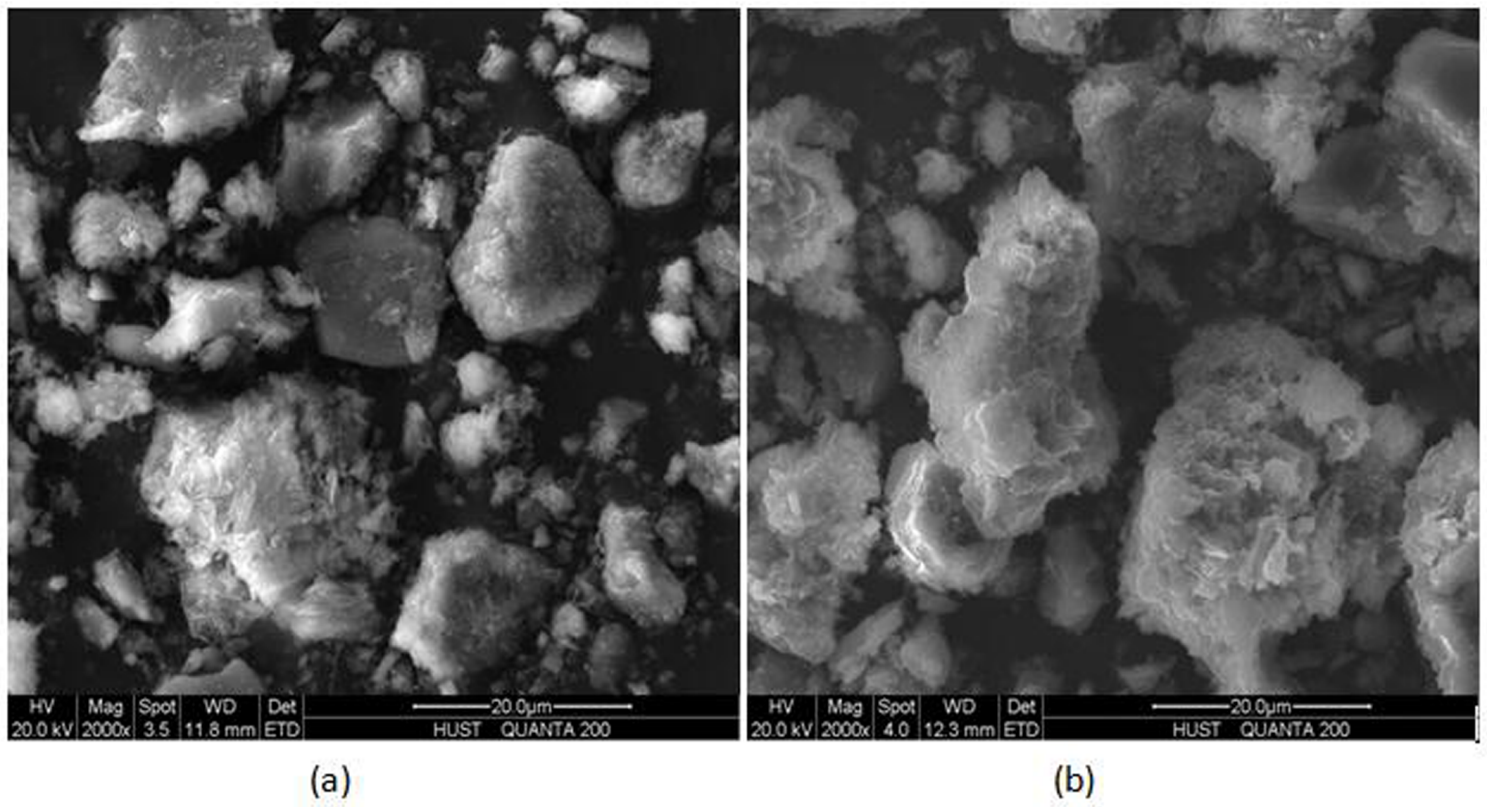
Scanning electron microscope (SEM) images of natural zeolite(a) and NaCl-zeolite (b).

Scanning Electron Microscope (SEM) images of natural zeolite and NaCl-modified zeolite were shown in Fig 3(a) and 3(b). Before modification, natural zeolite had an small pore size. After modification, the structure of the zeolite was looser than that of the natural zeolite. There were obvious changes in its original size, shape, crumb structure, and pore structure after NaCl modified which would improve adsorption capacity.

### Optimal conditions for NH_4_^+^-N and PO_4_^3−^-P removal by modified zeolite

pH is an important parameter in the batch adsorption study which will make influence on the adsorption process by changing the surface charge distribution of adsorbents used. While the dosage of zeolites can make influence on the adsorption process through changing unsaturation of the ion-exchange sites. A high adsorbent dosage can effectively decrease the unsaturation of the ion-exchange sites resulting in a lower ion exchange capacity[28].

#### Effect of pH

**Fig 4:**
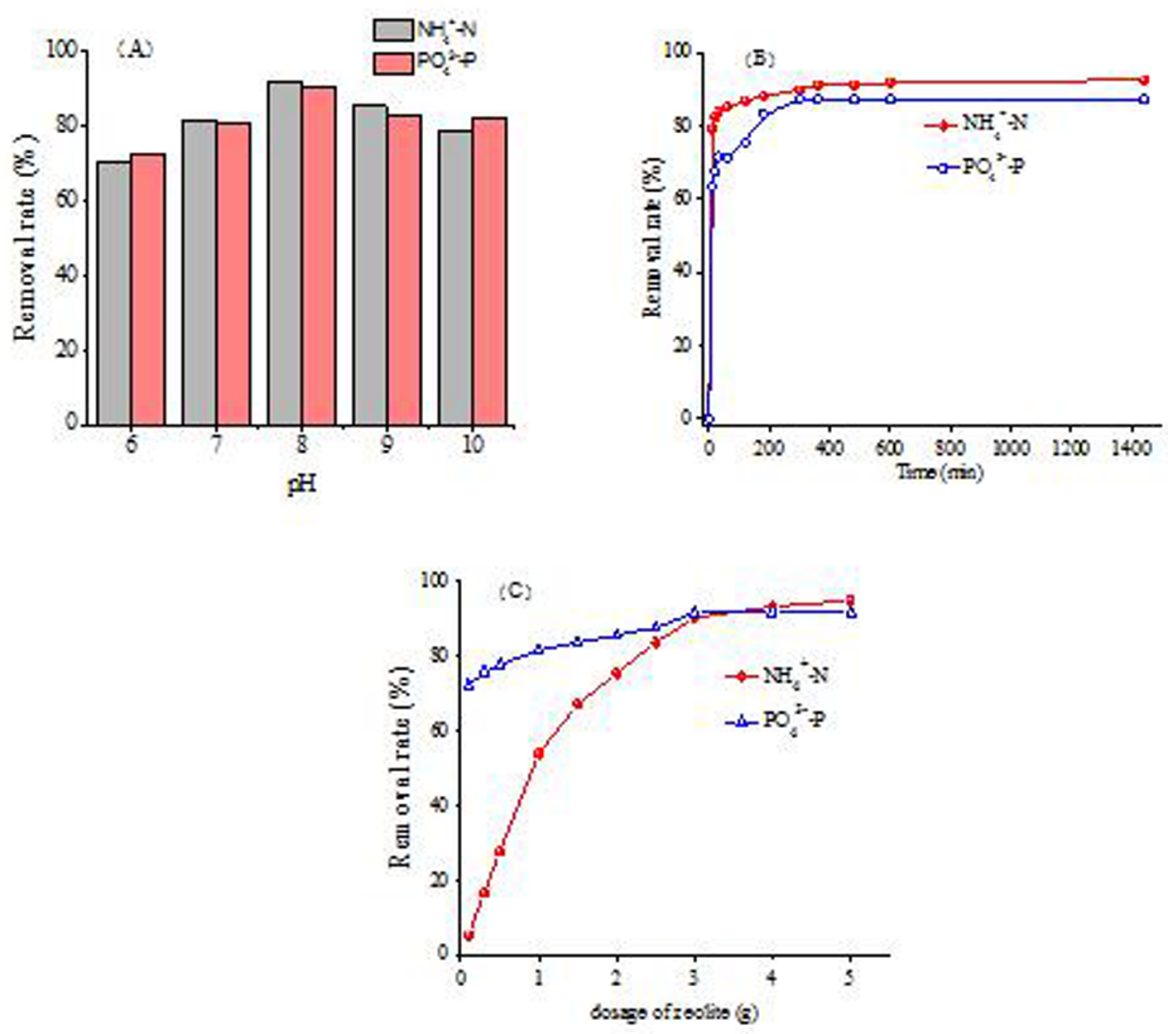
Optimal conditions for nutrients removal. (A)Effect of pH. (B) Effect of reaction time on removal efficiency of NH_4_^+^-N and PO_4_^3−^-P by NaCl-modified zeolite (1 g/100 ml). (C) Effect of dosage on removal efficiency of NH_4_^+^-N and PO_4_^3−^-P by NaCl-modified zeolite (1 g/100 ml).

To determine the optimal pH for NH_4_^+^-N and PO_4_^3−^-P removal, the ion-exchange performance of the MZ were investigated at different pH(from 6 to 10, (Fig 4(a)).

Result showed that MZ had the best removal efficiencies of NH_4_^+^-N(92.13%) and PO_4_^3−^-P (90.3%) were both occurred at pH 8. A higher or lower pH both would lead to negative results. The results were similar to Thornton and Marañón [29,30]. They proved an optimal range of pH 6–7 for NH_4_^+^-N remove by zeolite. However, Saltalı et al reported that the optimum NH_4_^+^-N remove efficiency was achieved at pH 8[31]. A favourable range of pH 4–8 for PO_4_^3−^-P remove by La/Al-modified zeolite was observed by Meng, while the PO_4_^3−^-P remove efficiency incresed with the decrease of pH varied from 3.0–10.0[32,33]. The inconsistency in the optimal pH may be attributed to the differences in the chemical compositions of the zeolites tested.

In the ion-exchange process, dissociated NH_4_^+^ will make the ion exchanged with Na^+^ in the zeolite. While the amount of NH_4_^+^ in the solution was depended on the pH of the solution. When pH is below 8, NH_4_^+^ ion concentration in solution rose with decrease of pH value, while H^+^ concentration increased with the drop in the pH which would had a negative influece on the NH_4_^+^ exchange [31]. When pH is over 8, NH_4_^+^ was converted into NH_3_ which not be exchanged on the zeolites [34]. The same as PO_4_^3−^-P remove efficiency, at relatively high pH values, OH^−^ concentration increased and competed with PO_4_^3−^-P on the adsorbent, leading to a lower phosphorous adsorption rate.

#### Effect of reaction time

Fig 4(b) showed the effect of the contact time on the removal efficiency of NH_4_^+^-N and PO_4_^3−^-P in biogas slurry at the dosage of 1 g zeolite/100 ml. It could be seen that both the NH_4_^+^-N and PO_4_^3−^-P removal efficiency increased with the contact time.

The concentration of NH_4_^+^-N and PO_4_^3−^P decreased rapidly during the first 0.5 h. The removal efficiency was 83.88% for NH_4_^+^-N and 71.49% for PO_4_^3−^P. Then in the following 5.5 h, corresponding NH_4_^+^-N and PO_4_^3−^-P removal efficiencies increased to 91.24% and 87.33%, respectively. Within 24 h, the concentration of NH_4_^+^-N decreased from 708.43 to 68.24 mg/L (92.58%) while the concentration of PO_4_^3−^-P decreased from 21.62 to1.81 mg/L (87.33% removal). However, with a further increase in the contact time from 30 min to 360 min, the NH_4_^+^-N removal efficiency increased slightly and then reached equilibrium after 360 min. This behavior could be attributed to the quick utilization of the most readily available adsorbing sites of the zeolites, leading to a fast diffusion and rapid attainment of equilibrium[35]. The increasing trend on nutrient sequestration over time (mainly within the first 6 h) is mainly related to the combined effect of dissociated Na_2_O and exchangeable ion on the zeolite surface (Table 3).

In order to analyze the adsorption performance of NH_4_^+^-N and PO_4_^3−^-P removal by zeolite, intra-particle diffusion model with three-linear regions was used according Eq (4):

$$q_{t}=k_{d}t^{1/2}+C$$ (4)

where k_d_ is the coefficient of intra-particle diffusion (mg g^-1^ h^-1/2^), t is the contact time(h).

Table 4 showed the results of kinetic parameters for ammonium and phosphate removal using the MZ. It indicated that the process of adsorption was more than one-step which could be validated by the prior experiments. It also conclued that surface diffusion adsorption process was very fast which was finished in a short time. The adsorption process of NH_4_^+^-N and PO_4_^3−^-P followed the second step of intra-particle diffusion model: surface diffusion adsorption process and particle diffusion adsorption process. Meanwhile the k_d1_value for NH_4_^+^-N was higher than k_d2_ and k_d3_ values which indicated that ammonium removal by zeolite was proposed as monolayer molecular adsorption with zeolite[36]. The k_d1_ and k_d2_ values for PO_4_^3−^-P was higher than k_d3_ value which indicated that PO_4_^3−^-P removal with zeolite was claimed as the electron exchange between phosphorus and the zeolite surface[37]. Moreover the k_d_ values for NH_4_^+^-N were both much higher than those for PO_4_^3−^-P, indicating that adsorption phase of P was very poor in the present zeolite, PO_4_^3−^-P and removal occurred most probably in the external boundary layer film of liquor surrounding the zeolite particles[38].

**Table 4:** Kinetic parameters for ammonium and phosphate removal using MZ.

Adsorption stage	Parameters	Intra-particle diffusion model		
		*C*_*i*_	*k*_*ip*_ (mg g^-1^ h^-1/2^)	*R*_*i*_^2^
first step	NH_3_-N	0.2157	1.0968	0.8734
	PO_4_^3−^-P	0.0067	0.0350	0.8807
second step	NH_3_-N	4.4353	0.0452	0.9250
	PO_4_^3−^-P	0.1422	0.0010	0.9114
third step	NH_3_-N	5.2200	0.0031	0.3440
	PO_4_^3−^-P	0.1583	0.0002	0.8915

#### Effect of zeolite dosages

Fig 4(c) showed the effect of adsorbent dosage on the NH_4_^+^-N and PO_4_^3−^-P removal. Both NH_4_^+^-N and PO_4_^3−^-P removal efficiency increased with the increase of the adsorbent dosage. The removal rates of NH_4_^+^-N increased from 5% to 95% with the increase of zeolite doses from 0.1 to 5.0 g/100 ml. The PO_4_^3−^-P removal rates also increased from 72% to 91% with zeolite doses ranging from 0.1 to 5.0 g/100 ml. Meanwhile when the dosage of zeolite increased from 0.1 to 3.0 g/100 ml, the NH_4_^+^-N removal efficiencies increased quickly from 5% to 90.37% compared with the PO_4_^3−^-P removal rates increasing from 72% to 91.63%. That was because the increasing amount of the adsorbent increased the surface area and the number of ion-exchange sites on zeolite. A negligible increase of NH_4_^+^-N and PO_4_^3−^-P removal efficiency occurred when the dosage was higher than 3.0 g/100 ml. This could be due to the fact that a high-adsorbent dosage can effectively decrease the unsaturation of the ion-exchange sites of the zeolites, and consequently, the number of such sites per unit mass gets reduced, resulting in comparatively lesser ion exchange at higher adsorbent amounts. Considerring the nutrient removal efficiency and economic cost, the optimum zeolites dose was 3.0 g/100 ml for the kind of zeolite on the removal of NH_4_^+^-N and PO_4_^3−^-P.

#### Adsorption isotherms and kinetic modelling

The parameters of two models calculated from the slope and the intercept of the plots are given in Table 5. Both two models could well described the adsorption isotherms process while the Langmuir model provided a slightly more consistent t to the data (R^2^:0.999 of NH_4_^+^-N and 0.992 of PO_4_^3−^-P) as compared with the Freundlich model (R^2^:0.905 of NH_4_^+^-N and 0.990 of PO_4_^3−^-P).

**Table 5:** Isotherms parameters of the Langmuir and Freundlich models.

Object	Langmuir parameter			Freundlich parameter		
	q_max_ (mg/g)	k_L_ (L/mg)	R^2^	k_F_ (mg/g)	1/n	R^2^
NH_4_^+^-N	11.25	0.035	0.999	1.26	0.36	0.905
PO_4_^3−^-P	6.67	0.79	0.992	3.1	0.31	0.990

In the present study, n values obtained between 2.78 and 3.23 which indicates a adsorption of NH_4_^+^-N, and PO_4_^3−^-P onto MZ. Langmuir adsorption capacity of NH_4_^+^-N, and PO_4_^3−^-P by MZ were compared with those of various low cost adsorbents as shown in Table 6. The maximum ion-exchange capacities of MZ at equilibrium (q_max_) were 11.25 and 6.67 mg/g respectively for NH_4_^+^-N and PO_4_^3−^-P. The differences of sorption capacity between various sorbents were caused by the difference in physico-chemical properties of adsorbents and experimental factors including the concentration range of NH_4_^+^-N, and PO_4_^3−^-P, pH, temperature, etc. Higher value of Langmuir constant and Freundlich adsorption capacity further reflect the improved strength and affinity of MZ for NH_4_^+^-N, and PO_4_^3−^-P.

**Table 6:** Comparison of Langmuir sorption capacity for NH_4_^+^-N and PO_4_^3^^−^-P with various adsorbents.

Adsorbent	Adsorption capacity (mg/g)		Conditions	Reference
	NH_4_^+^-N	PO_4_^3−^-P		
Bismuth impregnated biochar	---	1.48	pH 3.0, 318 k	[39]
Thermal activated sepiolite	2.93	---	pH 8.0, 303k	[40]
Raw fly ash	2.23	4.1	pH 7–8, 293k	[41]
Natural zeolite	3.45	---	pH 7, 298k	[42]
Chinese clinoptilolite	2.7–3.2	---	pH 6.0, 293k	[43]
NaCl- zeolite	11.25	6.67	pH 8.0, 298k	Present study

Adsorption for nutrients removal is a simple and the most economical method for wastewater treatment. The price and regeneration method are the main points when chosen as adsorbent. Modified NaCl-zeolite has serval traits that can be an ideal adsorbent.1) in comparison to the conventional adsorbent, it is very efficient. 2) it can be regenerated by chlorination regeneration easily. 3) the material is inexpensive, reducing the cost of wastewater treatment. 4) it does not contaminate the wastewater.

## Conclusion

In this research, simultaneous removal of NH_4_^+^-N and PO_4_^3−^-P from the effluents of biogas plants was investigated using NaCl-modified zeolite as an adsorbent material. The NaCl-modified zeolite showed a good absoprtion capacity to the NH_4_^+^-N and PO_4_^3−^-P which indicated that the proposed process for the treatment of digested swine wastewater is feasible. The condition of adsorption of NH_4_^+^-N and PO_4_^3−^-P onto NaCl-modified zeolite was pH 8.0, 3.0 g/100 ml and 6 h contact time.The adsorption isotherm of NH_4_^+^-N and PO_4_^3−^-P onto the adsorbent was well fitted to the Langmuir model and the maximum adsorption capacity (Qm) was 11.25 mg/g and 6.67 mg/g respectively.

## Supporting information

S1 Fig Fig 1 XRD patterns of the natural and modi ed zeolites. (XLSX)

S2 Fig Fig 2 FI-IR obtained for natural zeolite and NaCl-zeolite. (XLSX)

S3 Fig Fig 4 Optimal conditions for nutrients removal. (A)Effect of pH. (B) Effect of reaction time on removal efficiency of NH_4_^+^-N and PO_4_^3−^-P by NaCl-modified zeolite (1 g/100 ml). (C) Effect of dosage on removal efficiency of NH_4_^+^-N and PO_4_^3−^-P by NaCl-modified zeolite (1 g/100 ml).
(XLSX)
